# Knocking down LSD1 inhibits the stemness features of colorectal cancer stem cells

**DOI:** 10.1590/1414-431X20209230

**Published:** 2020-06-03

**Authors:** J. Chen, Jianyong Zhao, J. Ding, Ziwei Wang, Jiyi Du, Chenchang Wu

**Affiliations:** 1 Department of Gastrointestinal Surgery, Guizhou Provincial Bijie City Qixingguan District People's Hospital, Bijie, China; 2 Department of Gastrointestinal Surgery, Guizhou Provincial Staff Hospital, Guiyang, China; 3 Department of Gastrointestinal Surgery, Guizhou Provincial People's Hospital, Guiyang, China; 4 Department of Gastrointestinal Surgery, The First Affiliated Hospital of Chongqing Medical University, Chongqing, China; 5 Department of Gastrointestinal Surgery, The First People's Hospital of Guiyang, Guiyang, China

**Keywords:** Lysine-specific histone demethylase 1, Colorectal cancer, Stemness features

## Abstract

As a top leading cause of cancer death in many countries, colorectal cancer (CRC) has drawn increasing attention to the study of the pathological mechanism. According to the “cancer stem cell hypothesis”, malignancies originate from a small fraction of cancer cells that show self-renewal properties to initiate and sustain tumor growth and tumor metastasis. Therefore, these cancer stem cells (CSC) probably play important roles in tumor recurrence, metastasis, and drug resistance. Previous research reported that lysine-specific histone demethylase 1 (LSD1) maintains cancer stemness through up-regulating stemness markers SOX2 and OCT4. CD133 is believed to be the most robust surface marker for CRC stem cells, however the regulatory effect of LSD1 on stemness of CD133+ CRC has never been reported. In this study, our objectives included: 1) to isolate pure CD133+ and CD133− cells from SW620 cell line; 2) to investigate the effect of LSD1 on the characteristics of CD133+ stem cancer cells by knocking down the target gene. Results suggested that the SW620 cell line had both CD133+ and CD133− subsets. The CD133+ subset exhibited more CSC-like characteristics compared with the CD133− subset with higher viability, colony formation rate, migration and invasion rate, resistance to anti-cancer drugs, and apoptosis *in vitro*. The CD133+ also induced faster tumor formation and larger tumors *in vivo*. In the LSD1-knockdown CD133+ cells, the CSC-like characteristics had been all weakened. We conclude that LSD1 was important for CSCs to maintain their “stemness” features, which could be a potential therapeutic target of CRC.

## Introduction

As the third most prevalent cancer, colorectal cancer (CRC) is a major cause of mortality (1–3). In Asian countries, its incidence is increasing. According to WHO data, China had 245,000 new cases and 139,000 deaths of CRC in 2012 (4). In the trend study of CRC incidence and mortality in China, Zhang et al. (5) reported the increase of cancer cases from 104.3 thousand to 392.8 thousand from 1990 to 2019, as well as an increase of deaths from 81.1 thousand in 1990 to 167.1 thousand in 2016, predicting a further increase in the near future. At the time of diagnosis, 25% of newly diagnosed CRC patients will have metastases, commonly to the liver, lung, and peritoneum (6–8).

Fifty percent of all CRC patients die from metastatic disease (1–3). The “cancer stem cell hypothesis” has been proposed during recent years: a minority population of cancer cells (cancer stem cells, CSCs) within each tumor is able to initiate tumor growth (9,10). CSCs are tumor cells characterized with self-renewal, infinite proliferation, and potential of multi-directional differentiation properties (11,12). They are closely related to tumor metastasis, drug resistance, and recurrence after primary treatment. Recent microarray study supports the CSC model for metastases in epithelial tumors, including colon cancer (13,14). Additionally, some CSCs were reported with invasive and migratory phenotype required for the establishment and maintenance of metastatic disease early in their development (15).

The first convincing evidence of CSCs was reported by the identification of a subpopulation of leukemia cells expressing surface marker CD34 (16). Over time, colorectal CSCs were identified via a group of surface markers, including CD44, CD133, CD166, EPCM, and ALDH1 (11,17,18). Among those, CD133 is now believed to be the most robust surface marker for CRC stem cells (19). CD133 (known as prominin-1) was first discovered on normal human hematopoietic stem cells in 1997 (20,21). It is a five-transmembrane glycoprotein with a molecular weight of 120 kDa and shown to be mainly localized in membrane protrusions (22). CD133 antigen is expressed in colorectal cancer cell lines, such as HCT116, LOVO, HT29, and SW620 (23). Previous research reported that human colon cancer SW620 cells have both CD133+ and CD133− phenotypes: *in vitro*, CD133+ SW620 cells had a higher proliferative capacity and were more irradiation- and chemotherapy-resistant compared with CD133− cells (2). Additionally, injection of CD133+ cells induced larger tumors in mice compared to CD133−.

Recent advances in genomic and transcriptomic investigations revolutionized the field of oncology in the last decade and allowed the molecular profiling of thousands of tumors in different cancer types. By comparing metastases and surrounding tissue, various significantly upregulated proteins, such as lysine-specific histone demethylase 1 (LSD1/KDM1A), were identified. LSD1 is an epigenetic regulator that demethylates both the activating histone marker H3K4me and the repressive marker H3K9me (24). Overexpression of LSD1 facilitates proliferation, migration, invasion, and stemness of various malignancies, such as lung cancer (25), breast cancer (26), prostate cancer (27), hepatoma (28), and colon cancer (29). A high LSD1 expression was found to be significantly associated with tumor-node-metastasis (TNM) stages and distant metastasis (30). Research suggests that LSD1 possibly promotes metastasis of colon cancer by decreasing the level of demethylated histone H3 lysine4 (H3K4m2). Additionally, the deletion of LSD1 led to a reduced cell proliferation of colorectal cancer cells *in vitro* and *in vivo* (29). These findings led to the identification of LSD1 as a therapeutic target highly enriched in metastatic tissue. Currently, multiple LSD1 inhibitors have been developed for clinical trials (24).

Previous study confirmed that LSD1 regulates pluripotency of embryonic stem/carcinoma cells through up-regulating CSC markers SOX2 and OCT4 (31), however, its regulatory effect of LSD1 on stemness of CD133+ CRC has never been reported. In the present study, we sorted colon cancer cell lines SW620 to identify CD133+ and CD133− cells. Then, stemness was characterized on unsorted SW620 and sorted CD133+/CD133− cells. With more CSC-like characteristics, only CD133+ cells were used in the LSD1 knockdown studies. This study investigated the significance of LSD1 in tumorigenesis, especially in cell stemness, and provided a potential therapeutic target of colorectal cancer.

## Material and Methods

### SW620 cell sorting

Human colorectal cancer cell line SW620 was purchased from American Type Culture Collection (http://www.lgcstandards-atcc.org). The cells were maintained in 90% RPMI 1640 (Invitrogen, USA) medium supplemented with 10% fetal bovine serum (FBS). Cells were maintained at 37°C in a humidified environment of 5% CO_2_.

Cultured cell lines were isolated using the Diamond CD133 Isolation Kit (MACS, Miltenyi Biotec, Germany). When cell confluence reached 90% in the T75 flask, cells were digested and then suspended in the 200-μL buffer. Each suspension was incubated with 1 mL of Diamond Lin Biotin-Antibody Cocktail at 4°C for 10 min. Then, cells were rinsed with buffer, centrifuged at 825 *g* for 5 min at 27^o^C, followed by resuspension. Cells were mixed well with 100 μL CD133 Diamond MicroBeads at 4°C for 30 min. The mixture was then ready for stem cell separation on positive MACs separation (MS) sorting column in the magnetic field of a suitable MACS Separator. SW620 CD133− cells were collected from the effluent while SW620 CD133+ cells were first retained and then rinsed off from the MS sorting column. SW620 CD133− cells were then purified using the LD negative sorting column. All collected cells were counted and then the cell concentration was adjusted to 1×10^6^/mL. Cell suspension (1 mL) was washed with PBS, labelled with CD133 antibody (Alexa Fluor^®^ 488 conjugated #MAB4310X), and then incubated at 4°C for 30 min in the dark. Extra antibodies were removed by centrifugation (825 *g* for 5 min at 27°C) using 1 mL of PBS. Cells were resuspended in 200 μL PBS and tested on BD FACSCalibur. Results were recorded and analyzed in WinMD 12.9 software.

### Gene knockdown

The lentivirus system was used to knockdown LSD1 gene by transfecting SW620 CD133+ stem cells with LSD1-targeting shRNA. The infectious viruses (LV3-LSD1 and LV3-NC) were constructed by GenePharma (China). LV3-LSD1 was used to knockdown LSD1 gene with LSD1-targeting shRNA, while LV3-NC was used as negative control with scrambled control shRNA during transduction. The sequences used in virus construction were: 5′-CCGGATGACTTCTCAAGAA-3′ for LV3-LSD1 virus and 5′-TTCTCCGAACGTGTCACGT-3′ for LV3-NC virus. Viral titers were determined by GenePharma. Upon infection, the LVs were thawed on ice from -80°C freezer. SW620 CD133+ stem cells were cultured in 90% RPMI 1640 medium supplemented with 10% FBS, 100 U/mL penicillin, and 100 μg/mL streptomycin at 37°C in a humidified environment of 5% CO_2_. Single cell suspension was collected after trypsin treatment. The 10-cm dishes were coated with 0.001% poly-L-lysine for infection. When the cells were about 80% confluent, the medium was removed thoroughly, and 6 mL LV supernatants (LV3-LSD1 or LV3-NC) were added directly into the dishes. The cells were infected for 6 h or overnight. Then, the virus medium was replenished with 90% RPMI 1640 medium supplemented with 10% FBS.

### Western blotting

Transfected SW620 CD133+ stem cells were solubilized in RIPA lysis buffer (1% NP-40, 0.1% SDS, and 50 mM DTT) containing a cocktail of protease inhibitors (2 μg/mL aprotinin, 2 μg/mL leupeptin, and 1 mM PMSF). In addition, total protein from animal tissue was extracted using Total Protein Extraction Kit (Cat. No: SJ-200501, ProMab, China). Animal tissue (∼0.5 mg) was homogenized for 5∼20 min with 1 mL total protein extract buffer added and stilled on ice for 10∼20 min, followed by another 5∼20-min homogenization. Ultrasonic homogenization was performed three times for 3 s per time. The lysate was collected from the supernatant after centrifugation at 8568 *g* for 10 min, at 4°C. Proteins from lysates were separated on 5% PAGE-SDS gel (Sigma, China). Proteins and 5× loading buffer were mixed and boiled for 3 min for denaturation. In each well, 10∼20 μL of sample was loaded. After separating the protein mixture, it was transferred to a nitrocellulose blotting membrane (#88018, Pierce, China). The primary antibodies were first blocked for 2 h at room temperature. The secondary antibodies were then blocked after rinsing off the extra primary antibody and incubated overnight at 4°C. We used LSD1 (105 kDa, ab17721, Abcam, UK) and GADPH (37 kDa, SC-365062, SANTA, Santa Cruz, USA) primary antibodies, among which GADPH was the internal control. Secondary antibodies used in this study included: goat anti-rabbit IgG/HRP (1:1000 dilution) to probe LSD1 expression and goat anti-mouse IgG/HRP (1:800 dilution) to detect GADPH expression. The protein bands were detected using PBST (phosphate buffered saline with Tween^®^ 20 detergent) and visualized in the dark room. The positive bands were analyzed by Gel-Pro Analyzer (version 4.0; Media Cybernetics, USA) and their integrated absorbance (abs) values were measured.

### *In vitro* colony formation assay

A 2-D *in vitro* culture assay was used to test the cell ability of colony formation. A monolayer of tested SW620 cells in the logarithmic growth phase was scattered with 0.25% trypsin into individual cells. One thousand cells were seeded in sterile 6-cm-diameter plates. The cell suspension was inoculated separately into a plate with 3 mL of 37°C pre-warmed culture solution. Cultures were gently rotated to uniformly disperse cells. Then, the cells were cultured at 37°C in a humidified environment of 5% CO_2_ for 2-3 weeks. Colonies were fixed with methanol for 20 min and stained with Giemsa. Visible colonies were then counted to calculate the colony formation rate using the following formula: Colony formation rate = number of colonies / number of inoculated cells × 100%.

*In vitro* colony formation assay was performed on SW620 CD133−, SW620 CD133+, as well as oxaliplatin IC50-treated SW620 CD133−, SW620 CD133+, and LSD1-knockdown SW620 CD133+ cells (see below for details of oxaliplatin IC50).

### MTT assay

Unsorted SW620 cells, CD133+, and CD133− cells were cultured as described above and then treated with anti-cancer drugs as described below. Cells (approximately 1×10^4^) in 100 μL of culture medium were plated onto 96-well plates and incubated at 37°C in a humidified environment of 5% CO_2_ for 24 h. A control of cells in medium and background of medium alone were also plated. Cells in treated groups were then exposed to varying concentrations of the drug for 48 h. Six concentrations of oxaliplatin (0.25, 0.5, 1, 2, 4, and 8 μM) and 5-fluorouracil (5-FU) (0.5, 1, 2, 5, 10, and 20 μg/mL) were used. After exposure to the drug, the cells were treated with 50 μL diluted 1×MTT (3-(4,5-dimethylthiazol-2-yl)-2,5-diphenyl tetrazolium bromide) dye and incubated for 4 h at 37°C. All liquid was then removed with a needle and syringe. Then, 150 μL of dimethyl sulfoxide (DMSO) was added to each well to dissolve the MTT-formazan crystals.

The plates were positioned in a scanning multi-well spectrophotometer (MR5000; Dynatech Laboratories Inc., USA). Each plate was then photometrically quantified at an absorbance of 570 nm. The amount of purple formazan produced by treated cells was compared with that of the untreated control cells, and the absorbance in the treated cells (abs_treated cells_ – abs_medium background_) is reported as a percentage of control: Cell viability (%) = (abs_treated cells_ – abs_medium background_) / (abs_control_ – abs_medium background_) × 100%.

Half maximal inhibitory concentration (IC50) of both oxaliplatin and 5-FU was calculated according to the cell viability of unsorted SW620 CD133 cells. The viability was investigated on SW620 CD133−, SW620 CD133+, as well as IC50-treated SW620 CD133−, SW620 CD133+, and LSD1-knockdown SW620 CD133+ cells.

### Cell migration assay

Cells in logarithmic growth phase were plated in 6-well plates. When cell confluency reached more than 80%, a horizontal line was drawn at the bottom of each plate using 200-μl pipette tips. The plates were then rinsed three times with PBS to remove cells that had peeled off. After the above-mentioned treatment, the distance of cells was determined by Image-Pro Plus 6.0 software (Media Cybernetics Inc.) and the cell migration rate was calculated by the following formula: cell migration rate (%) = [(initial distance – final distance) / initial distance] × 100%.

### Cell invasion assay

Cell invasion was evaluated using a BD BioCoat Matrigel invasion chambers (Becton-Dicknson). Cells were seeded in soft-agar plates in the upper chamber (100 μL/well) and then incubated at 37°C in 5% humidified CO_2_ for 24 h. Cell invasion was determined by measuring the area occupied by the cells on the lower side of the filter. The area occupied by the cells that passed through the membrane was calculated under a microscope in 10 randomly selected fields at 200× magnification using the public domain ImageJ program (NIH, USA). The measurement was repeated in three specimens.

### Apoptosis assessment

Annexin V-FITC Apoptosis Detection Kit (Sigma-Aldrich, China) was used in accordance with the manufacturer's protocol. After treatment of cells with 0.3 μg/mL doxorubicin (Adriblastina^®^ Pharmacia; Slovakia) for 16 h, the cells were washed with PBS twice, resuspended in binding buffer, and incubated with 1 μL annexin V-FITC and 2 μL PI in 100 μL binding buffer for 10 min in the dark at room temperature. Then, the cells were analyzed by flow cytometry (ATTUNE^®^ Flow cytometer (Applied Biosystems, China), Stem Cell Technology Research Center). Apoptosis assessment was performed on SW620 CD133−, SW620 CD133+, as well as IC50-treated SW620 CD133−, SW620 CD133+, and LSD1-knockdown SW620 CD133+ cells.

### *In vivo* animal assays

Tested cells were maintained in 90% RPMI 1640 medium supplemented with 10% FBS for future use, and passaged every 3 days. The animal experiment was approved by the Ethical Committee for Animal Research of Southern Medical University (protocol number: 2011-020). BALB/c nude mice (females, 4-6 weeks old) were purchased from the Central Animal Facility of Southern Medical University. To generate tumor growth *in vivo*, 1×10^6^ cells (0.2 mL cell suspension per inoculation site) were subcutaneously injected into limb-adjacent tissues where tumor forms more easily. All mice were kept under specific pathogen-free conditions with a 12-h light/dark cycle and autoclaved food/water were provided freely. When tumors were readily observed (approximately one week after injection), the longest diameter “a” and the shortest diameter “b” of tumors were measured every 3 days. The tumor volume was calculated using the formula: tumor volume (mm^3^) = a × b^2^ × 52%. The tumor growth curve was plotted with time as the abscissa and tumor volume as the ordinate. All mice (3 mice were tested for each treatment/control group) were sacrificed after 25 days, and the removed tumors were weighed and photographed. *In vivo* animal assay was performed on SW620 CD133−, SW620 CD133+, as well as IC50-treated SW620 CD133−, SW620 CD133+, and LSD1-knockdown SW620 CD133+ cells (see below for details of oxaliplatin or 5-FU IC50).

### Statistical analysis

All tests were carried out in triplicate. Data means were compared using Student's *t*-test. Statistical analyses were performed by SPSS version 16.0 analytical software (IBM, USA). Statistical significance was defined as P<0.05.

## Results

### Characteristics of SW620 stem cells

**Figure 1:**
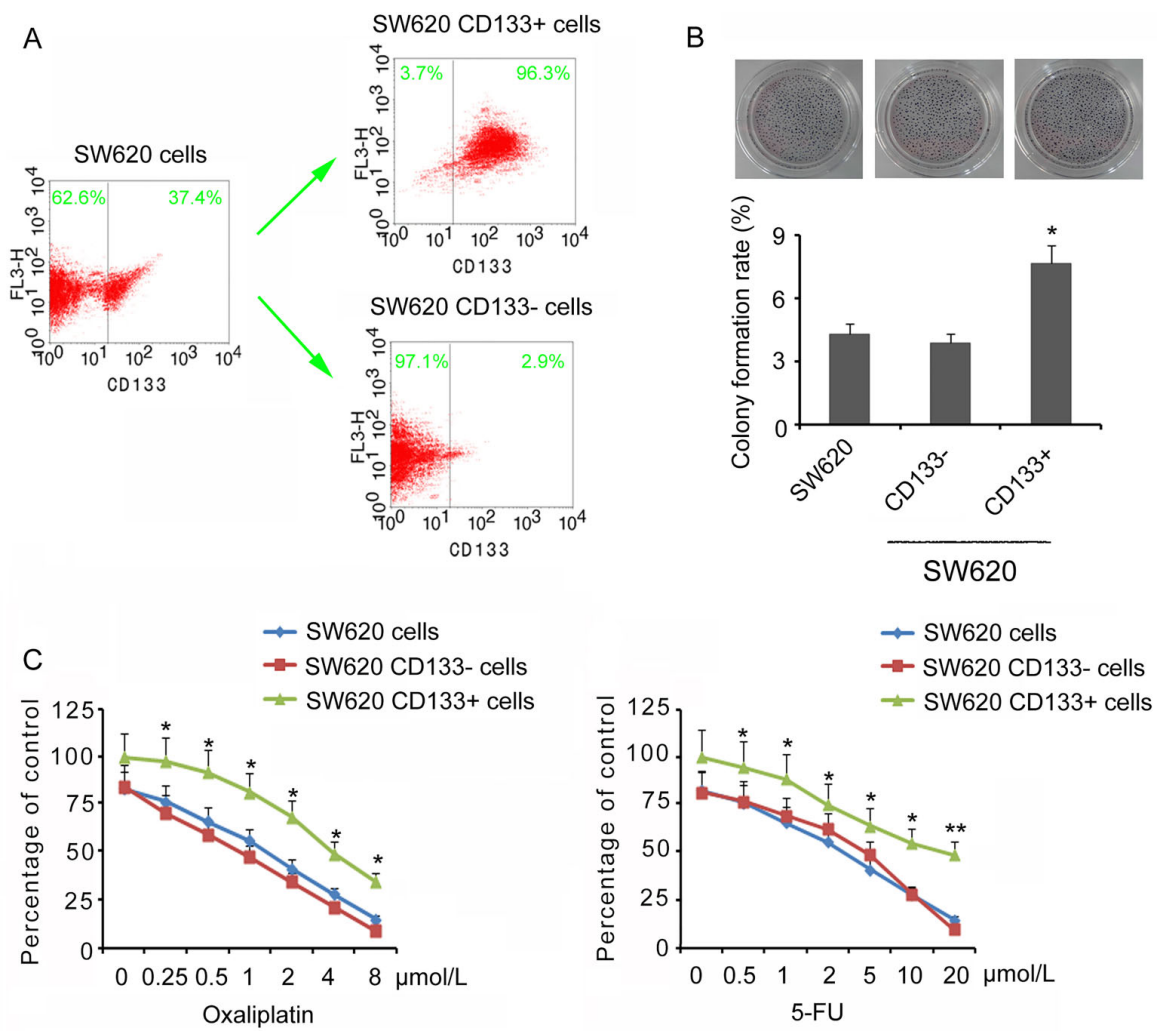
Characteristics of SW620 stem cells. **A**, SW620 CD133+ and CD133− cells were isolated from SW620 using flow cytometry. **B**, Colony formation of unsorted SW620, CD133−, and CD133+ cells. **C**, Relative cell viability of unsorted SW620, CD133−, and CD133+ cells under oxaliplatin and 5-FU (5-fluorouracil) treatments. Data are reported as means±SD. *P<0.05 and **P<0.01 *vs* unsorted SW620 cells (*t*-test).

SW620 CD133+ and CD133− cells were isolated from SW620 using flow cytometry (Figure 1A).

The “stemness” of unsorted SW620 cells, and CD133+ and CD133− cells were tested by growing single cells in soft agar (2-D culture). In all cases, colonies were visible after two weeks but the number of colonies was significantly greater in CD133+ cells compared to unsorted SW620 cells and CD133− cells (P<0.05, Figure 1B). The resistance of unsorted SW620 cells, CD133+, and CD133− cells was tested when exposed to anti-cancer drugs at different levels. Concordant results were obtained from both oxaliplatin and 5-FU treatments: CD133+ cells had a significantly higher viability rate under exogenous stress than unsorted SW620 cells, while no significance was observed comparing CD133− cells versus unsorted SW620 cells (Figure 1C). From the curves, IC50 of oxaliplatin and 5-FU for different cells were determined: IC50 of oxaliplatin was 1.875 μM for unsorted SW620 cells, 1.64 μM for CD133− cells, and 4.04 μM for CD133+ cells, while 5-FU was 2.87 μM for unsorted SW620 cells, 2.45 μM for CD133− cells, and 3.93 μM for CD133+ cells. In the following chemotherapy treatments, the IC50 of oxaliplatin (1.875 μM) and 5-FU (2.87 μM) of unsorted SW620 cells were used in CD133− and CD133+ cells to determine their difference in response to both oxaliplatin and 5-FU.

### LSD1 expression was higher in CD133+ cells than unsorted SW620 cells and CD133− cells

**Figure 2:**
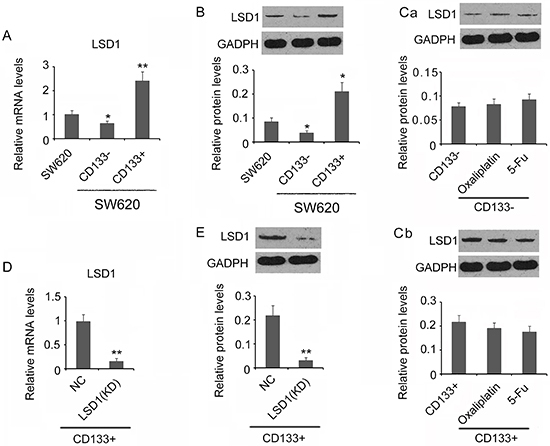
Quantified LSD1 mRNA and LSD1 protein in different cells. **A**, Relative LSD1 mRNA and protein level (**B**) in unsorted SW620, CD133−, and CD133+ cells. GADPH is the internal control. **Ca** and **Cb**, Relative LSD1 protein level in CD133− and CD133+ cells under oxaliplatin and 5-FU IC50 treatment. GADPH is the internal control. **D**, Relative LSD1 mRNA in non-treatment control (NC) and LSD1 knockdown CD133+ cells (KD). **E**, Relative LSD1 protein level in NC control and LSD1 knockdown CD133+ cells (KD). Data are reported as means±SD. **A** and **B**, *P<0.05 and **P<0.01 *vs* SW620 cells; **C**, no significance; **D** and **E**, **P<0.01 *vs* NC (*t*-test).

In previous reports, overexpression of LSD1 promoted proliferation, migration, and invasion of various malignancies (32–34). Significantly higher mRNA and more LSD1 protein were observed in CD133+ cells compared to unsorted SW620 cells (P<0.05 or P<0.01, Figure 2A and B). Conversely, CD133− cells expressed significantly less LSD1 mRNA and LSD1 protein compared to unsorted SW620 cells (P<0.05, Figure 2A and B). In both CD133− and CD133+ cells, usage of anti-cancer drugs, either oxaliplatin or 5-FU, did not change the LSD1 protein content, indicating the commonly used drugs are not targeting LSD1 for chemotherapy (Figure 2C). As the major stem cells in SW620 cells, CD133+ was selected in LSD1 knockdown. SW620 CD133+ cells were transfected with either LSD1 specific (LV3-LSD1) or scramble control shRNA (LV3-NC) and were tested post-overnight transfection. In LSD1 shRNA-transfected CD133+ cells (knockdown, KD), the LSD1 mRNA and LSD1 protein were both significantly lower than NC cells, indicating the successful reduction of LSD1 expression in CD133+ cells (P<0.01, Figure 2D and E).

### LSD1 knockdown impaired the “stemness” of CD133+ cells

**Figure 3:**
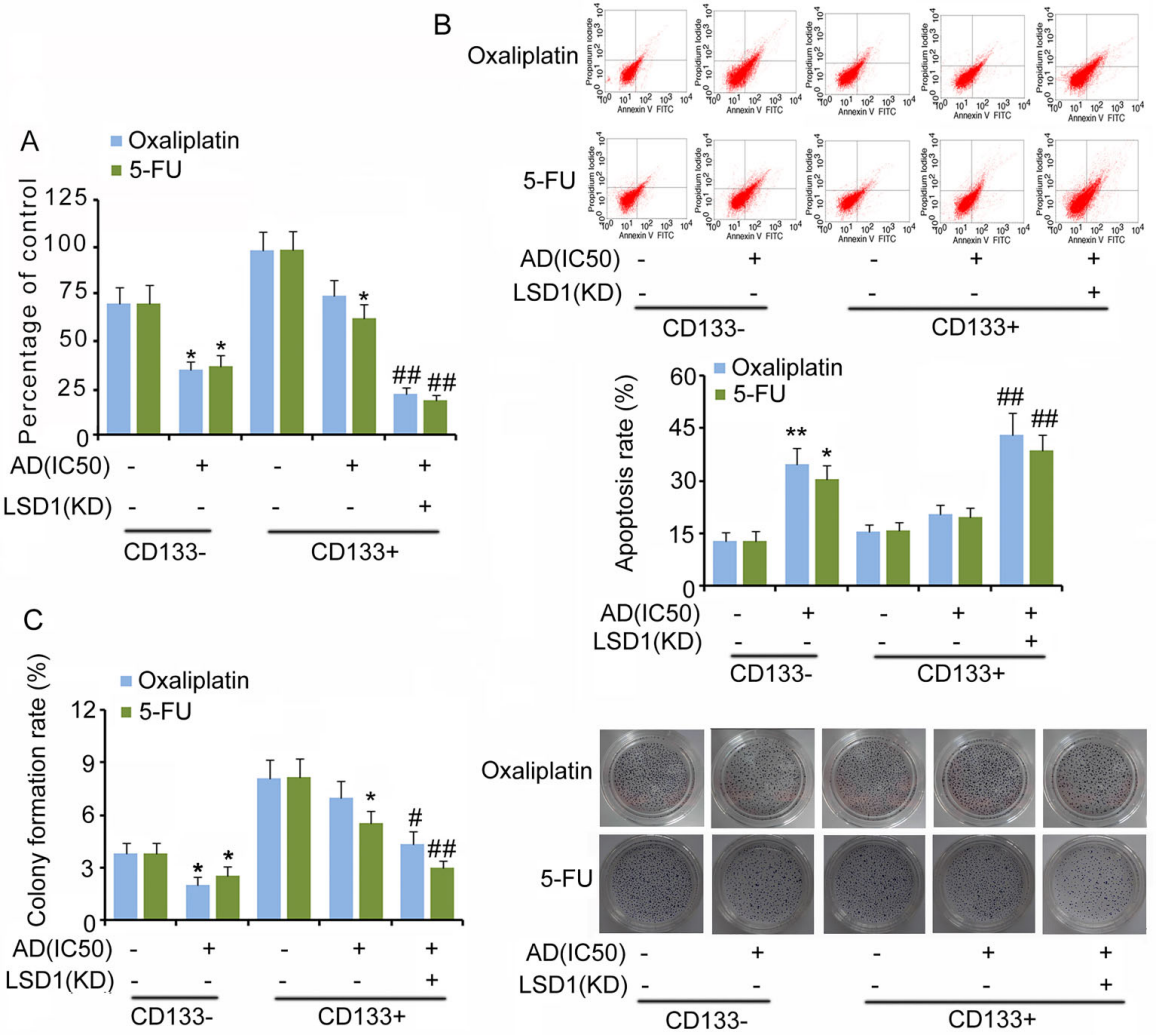
Cell viability, apoptosis, and colony formation of CD133−/CD133+ in response to anti-cancer drugs. **A**, Relative cell viability, **B**, apoptosis rate, and **C**, colony formation rate of CD133−/CD133+ and LSD1-KD CD133+ cells. Oxaliplatin and 5-fluorouracil (5-FU) were dissolved using cell culture medium. The untreated groups that were used to compare to oxaliplatin and 5-FU groups are the same. Data are reported as means±SD. *P<0.05 and **P<0.01 *vs* non-treatment groups; ^#^P<0.05 and ^##^P<0.01 *vs* AD treatment groups (*t*-test). AD: anti-cancer drugs; IC50: half maximal inhibitory concentration; KD: knockdown.

**Figure 4:**
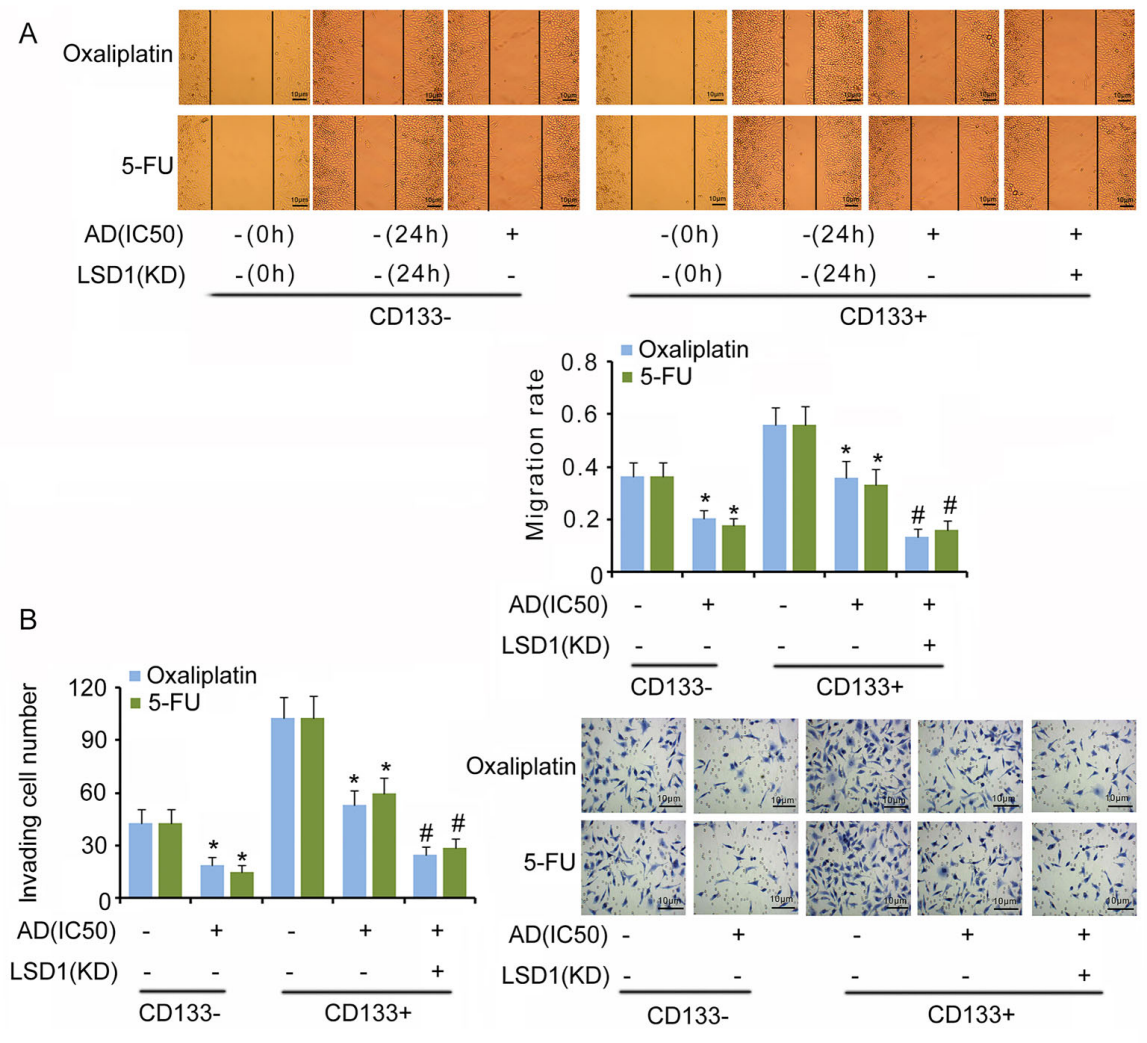
Cell migration (**A**) and invasion (**B**) of CD133−/CD133+ and LSD1-KD CD133+ cells in response to anti-cancer drugs. Oxaliplatin and 5-fluorouracil (5-FU) were dissolved using cell culture medium. The untreated groups that were used to compare to oxaliplatin and 5-FU groups were the same. Data are reported as means±SD. *P<0.05 *vs* non-treatment groups; ^#^P<0.05 *vs* AD treatment groups (*t*-test). AD: anti-cancer drugs; IC50: half maximal inhibitory concentration; KD: knockdown.

Results indicated that both anti-cancer drugs oxaliplatin and 5-FU at IC50 significantly decreased cell viability (P<0.05, Figure 3A), colony formation (P<0.05, Figure 3C), migration (P<0.05, Figure 4A), and invasion (P<0.05, Figure 4B) of CD133− cells, but increased the apoptosis rate (P<0.05 or P<0.01, Figure 3B) compared to those in untreated cells. However, oxaliplatin at IC50 only inhibited the migration and invasion of CD133+ cells (P<0.05); 5-FU at IC50 significantly inhibited the cell viability, colony formation, migration, and invasion of CD133+ cells (P<0.05). Both oxaliplatin and 5-FU had no effect on CD133+ cell apoptosis. LSD1 knockdown further decreased cell viability, colony formation rate, migration, and invasion of CD133+ cells in response to oxaliplatin and 5-FU, and increased the apoptosis rate (P<0.05 or P<0.01).

**Figure 5:**
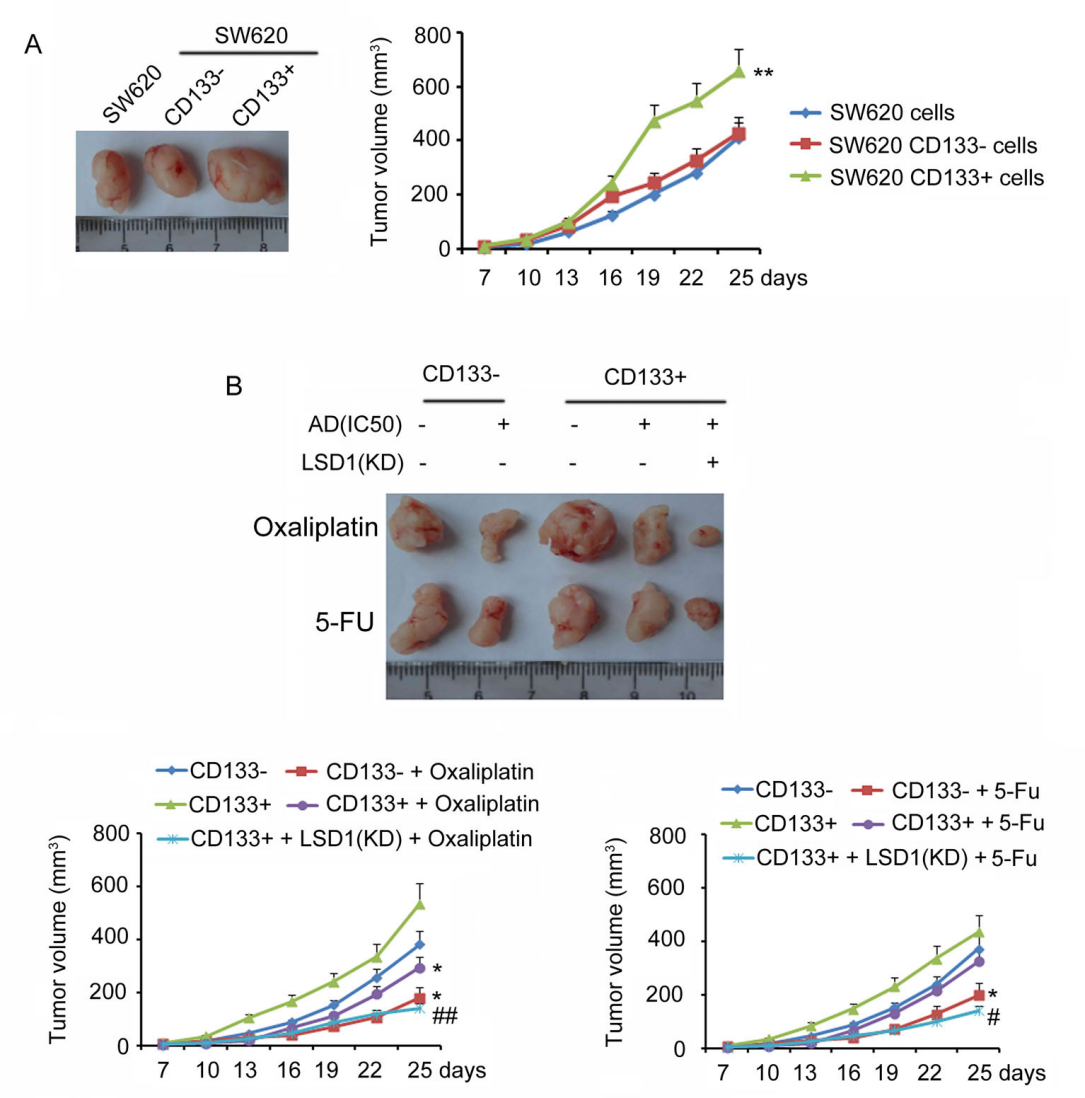
Tumor formation *in vivo* animal assays. **A**, Tumor volume induced by unsorted SW620, CD133−, and CD133+ cells from days 7 to 25 after cell injection. **B**, Tumor volume induced CD133−/CD133+ and LSD1-KD CD133+ cells under oxaliplatin or 5-fluorouracil (5-FU) treatment. Data are reported as means±SD. **A**, **P<0.01 *vs* SW620 cells; **B**, *P<0.05 *vs* non-treatment groups; ^#^P<0.05 and ^##^P<0.01 *vs* AD treatment groups, (*t*-test). AD: anti-cancer drug.

Additionally, cell stemness was evaluated *in vivo*. The tumor volume was observed from days 7 to 25 after the injection of unsorted SW620, CD133−, and CD133+ cells. CD133+ cells started to induce faster tumor formation and larger tumors after 16 days (P<0.01, Figure 5A). However, no difference in tumor formation or tumor volume was observed between unsorted SW620 cell treatment and CD133− cell treatment. In order to better evaluate the effect of LSD1 to the stemness of CD133+ cells, IC50 of oxaliplatin and 5-FU were used to study the chemotherapy-resistance of CD133− cells as well as CD133+ cells with LSD1 knockdown or not. Oxaliplatin and 5-FU inhibited the tumor formation of CD133− cells (P<0.05, Figure 5B). In contrast, the tumor formation of CD133+ cells was only inhibited by oxaliplatin (P<0.05). CD133+ cells with LSD1 knockdown showed slower tumor formation and smaller tumor volume than CD133+ cells (P<0.05 and P<0.01).

## Discussion

It has been reported that some leukemia cells have tumorigenic potential, and that these could be distinguished by surface markers (32,33). Regarding human CRC, there are studies that identified and separated CSCs using CD133 as a marker (17–19). Therefore, in the present study, we tested the SW620 cell line to investigate the CSC-like characteristics of CD133+/CD133− cells with regard to cell viability, colony formation, migration and invasion, apoptosis-resistance, chemotherapy-resistance *in vitro*, and tumor formation *in vivo*.

*In vitro*, we found that CD133+ SW620 cells exhibited higher viability, colony formation, migration and invasion, and lower apoptosis compared with unsorted SW620 and CD133− SW620 cells, which were concordant with the characteristics of CSCs. Consistently, CD133+ cells induced faster tumor formation and larger tumors *in vivo*. Our result confirmed again that CD133+ have tumorigenic potential (2,35); however, our results did not show a significant difference between CD133− and unsorted SW620 cells. Though the tumorigenic potential of CD133− cells was also reported in the glioma cell line (36), the CD133+ subset exhibited more CSC-like characteristics compared with the CD133− subset.

The responses of CD133+ and CD133− SW620 cells to anti-cancer drugs were not completely consistent in this study. Both oxaliplatin and 5-FU inhibited the cell viability, colony formation, migration, and invasion of CD133− SW620 cells and increased apoptosis. Although 5-FU at the same dosage also inhibited the cell viability, colony formation, migration, and invasion of CD133+ SW620 cells, it had no significant effect on the apoptosis. Oxaliplatin only inhibited the migration and invasion of CD133+ SW620 cells; it had no significant effects on the cell viability, colony formation, and apoptosis. All results suggested that CD133+ SW620 cells have stronger resistance to oxaliplatin and 5-FU than CD133− SW620 cells.

This study tested the effects of LSD1 on the response to anti-cancer drugs and maintenance of the “stemness” characteristics of CD133+ cells. In LSD1-knockdown CD133+ cells, both oxaliplatin and 5-FU performed extremely well in breaking down all stemness characteristics of CD133+ cells either *in vitro* or *in vivo*: silencing of LSD1 gene effectively inhibited the viability, colony formation, and invasion of colon cancer cells. In a previous study, it is reported that LSD1 is closely related to the metastasis of colon cancer. The expression of LSD1 in epithelial-derived tumors such as breast cancer, lung cancer, bladder cancer, liver cancer, and even mesenchymal tumors was significantly upregulated (37). Upregulation of LSD1 induced tumor formation through chromatin modification (38), inhibited tumor cell apoptosis by removing methylation modification at 370 lysine of p53 (39), and promoted tumor cell proliferation. Inactivation of LSD1 inhibited the expression of stem cell markers Oct4 and Sox2 (31). The usage of sodium arsenite reduced the viability and stemness of CD133+CD13+ hepatocytes and attenuated the tumorigenicity of CD133+CD13+ hepatocytes xenografts in mice. The effects of sodium arsenite were mediated by the inhibition of Oct4, Sox2, and Klf4 expression (40). Though oxaliplatin and 5-FU are commonly used anti-cancer drugs, neither of them changed the content of LSD1 protein. Therefore, LSD1 may be a new target for chemotherapy in cancer stem cells through targeting the CSC stemness.

In conclusion, we found that a human colon cancer cell line, SW620, has both CD133+ and CD133− subsets and that the CD133+ subset exhibited more CSC-like characteristics compared with the CD133− subset. We propose LSD1 as a potential therapeutic target in future studies.
